# Technology-enhanced compression and AI-integrated lymphedema care: a narrative review

**DOI:** 10.1007/s11845-025-04101-4

**Published:** 2025-11-03

**Authors:** Amany Gomaa Atiaa, Mostafa M. Mostafa, Doha El-Sayed Ellakwa

**Affiliations:** 1 Department of Physical Therapy for Surgery, Faculty of Physical Therapy, Sinai University, Kantra Branch, Ismailia, Egypt; 2 Department of Molecular and Cellular Physiology, Stritch School of Medicine, Loyola University Chicago, Chicago, USA; 3 Department of Biochemistry and Molecular Biology, Faculty of Pharmacy for Girls, Al-Azhar University, Cairo, Egypt; 4 Department of Biochemistry, Faculty of Pharmacy, Sinai University, Kantra Branch, Ismailia, Egypt

**Keywords:** Artificial intelligence (AI), Compression therapy, Digital health, Lymphedema, Mobile health apps, Physical therapy, Rehabilitation, Telehealth, Wearable technology

## Abstract

**Background:**

Lymphedema is a persistent and often debilitating condition resulting from lymphatic system dysfunction. It frequently develops as a secondary complication, especially after cancer treatments. Traditional management, centered on complete decongestive therapy (CDT), offers modest benefits but is limited by accessibility, long-term adherence, and variable patient response.

**Aims:**

This narrative review explores how digital health and enhanced compression technologies are reshaping lymphedema care.

**Methods:**

A comprehensive review of pertinent literature was conducted to assess the utilization of artificial intelligence (AI), telehealth platforms, mobile applications, virtual reality-based rehabilitation, and advanced compression devices in the management of lymphedema. Focus was directed towards their clinical efficacy, patient-centered advantages, and the challenges associated with their implementation.

**Results:**

AI-driven instruments exhibit significant promise for precise risk stratification, prompt diagnosis, and individualized treatment planning. Digital platforms—including telehealth services, mobile applications, and virtual rehabilitation programs—improve accessibility, enhance patient engagement, and promote sustained adherence over time. Cutting-edge compression modalities, such as sensor-equipped garments, adaptive pneumatic systems, and vibration-assisted apparatus, deliver dynamic, feedback-informed therapeutic interventions. The amalgamation of these technological advancements with wearable biosensors and AI-powered platforms has yielded promising outcomes in limb volume regulation, rehabilitation efficacy, and overall quality of life. Nevertheless, challenges persist concerning equitable access, compliance with regulatory frameworks, cost-effectiveness, and the preparedness of clinicians.

**Conclusion:**

Digital health and sophisticated compression methodologies signify a transformative advancement in lymphedema management, promoting anticipatory, individualized, and universally accessible healthcare. Mitigating existing obstacles is crucial for the effective incorporation of these approaches into standard clinical protocols.

**Graphical Abstract:**

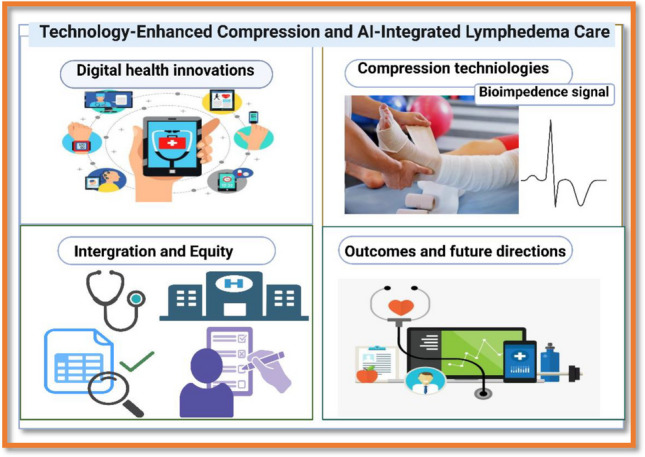

## Introduction

Lymphedema is a chronic, progressive disorder resulting from the mechanical failure of the lymphatic system to drain protein-rich interstitial fluid effectively [1]. This dysfunction leads to persistent tissue swelling, dermal fibrosis, and significant impairments in physical function [2, 3]. Clinically, lymphedema is classified as either primary—stemming from congenital or genetic anomalies of lymphatic architecture—or secondary, which arises from acquired disruptions such as cancer surgery, lymphadenectomy, radiation therapy, trauma, infection, or systemic disease [4–7]. Secondary lymphedema represents the vast majority of cases and is especially prevalent among individuals undergoing oncologic treatments for breast, gynecologic, prostate, and head and neck cancers [8, 9]. The epidemiological burden of secondary lymphedema is substantial yet varies by cancer type, therapeutic protocol, and geographic setting. For instance, the prevalence of upper-limb lymphedema following breast cancer treatment ranges from 3 to 87%, with most estimates converging between 20 and 49% over a 10-year period [10–14]. In low- and middle-income countries, lower-limb lymphedema occurs in approximately 10% of women post-gynecologic cancer therapy, while nearly 29% of prostate cancer survivors receiving combined radiation and surgery report genital or lower-limb lymphedema [15]. Head and neck cancer survivors present with some of the highest rates, exceeding 75%, often involving both external and internal edema that severely compromises function and quality of life [15],Ellakwa D et al., [16]). Nevertheless, true prevalence is likely underreported due to heterogeneity in diagnostic criteria, inconsistent measurement techniques, and data capture limitations. U.S. insurance claims, for example, estimate an incidence of just 0.95% to 1.24% across all cancer types [17, 18]. Beyond the physical sequelae—such as limb heaviness, restricted mobility, and heightened infection risk—lymphedema imposes a substantial psychosocial burden [19]. Affected individuals frequently experience anxiety, depression, stigma, and social withdrawal, further diminishing functional capacity, social participation, and quality of life [20],“Overview of Lymphedema for Physicians and Other Clinicians,” 2022; [7, 8]. The cornerstone of current non-surgical management is complete decongestive therapy (CDT), which integrates manual lymphatic drainage (MLD), compression therapy, targeted exercise, and meticulous skin care. While MLD techniques facilitate lymph flow and offer moderate reductions in limb volume, their long-term efficacy is variable [9, 13]. Compression garments and multilayer bandages are central to edema maintenance, and therapeutic exercise supports lymphatic return and mobility [21]. Rigorous skin hygiene is also critical to prevent recurrent infections such as cellulitis (Ellakwa D et al., [22],“Overview of Lymphedema for Physicians and Other Clinicians,” 2022. Despite CDT's central role, several barriers impede its effectiveness: inconsistent protocols, limited standardization of diagnostics, poor long-term adherence, and uneven access to trained therapists [23–25]. Surgical options such as lymphovenous bypass and vascularized lymph node transfer offer hope for advanced or refractory cases, yet their adoption is constrained by cost, availability, and procedural complexity (De Senna Nogueira Batista & Chang, [26–28],). Furthermore, self-management approaches are often psychologically burdensome and inadequately supported by health systems, particularly in underserved settings [29, 30].

In response to these unmet clinical needs, a paradigm shift is underway—toward patient-centered, technology-enabled models of care [31]. Emerging innovations such as artificial intelligence (AI)**–**based risk stratification, telehealth-delivered rehabilitation, and sensorized compression technologies promise to enhance early detection, personalize treatment, and improve adherence across diverse populations [4–6, 27, 28, 32]. These convergent technologies herald a new era of lymphedema management—more predictive, participatory, and equitable in scope [33, 34].

## Study design and scope

This review adopts a narrative synthesis methodology to evaluate recent innovations in the physical therapy management of secondary lymphedema. It focuses specifically on the integration of digital health technologies and next-generation compression systems. The objective is to critically appraise emerging modalities that may enhance patient outcomes, improve adherence, and expand accessibility relative to traditional care models.

### Literature search strategy

A structured literature search was conducted across four major electronic databases—PubMed, Scopus, Web of Science, and Google Scholar—to identify relevant publications from January 2016 to June 2025. The search strategy combined keywords and Medical Subject Headings (MeSH), including: “lymphedema,” “complete decongestive therapy,” “digital health,” “telehealth,” “artificial intelligence,” “compression therapy,” “wearable devices,” and “rehabilitation technology.” These terms were combined with corresponding MeSH terms to enhance the accuracy and comprehensiveness of the search.

### Inclusion and exclusion criteria

Studies were included if they met the following criteria:

- Focused on physical therapy interventions for secondary lymphedema, particularly following oncologic treatment.
- Explored digital or technological innovations, such as telehealth platforms, mobile applications, virtual reality (VR) tools, or advanced compression devices (e.g., sensorized garments, pneumatic sleeves).
- Reported empirical outcomes, including limb volume reduction, adherence rates, usability, or device performance.

Studies were excluded if they:

- Focused exclusively on primary lymphedema.
- Were not published in English**.**
- Consisted only of editorials, expert opinions, or abstracts without empirical data**.**

“Studies not published in English were excluded due to potential language barriers that may affect the accuracy of translation and interpretation of study findings. Additionally, to ensure consistency in data analysis and quality assessment, English was set as the primary language criterion for the review, as the majority of relevant literature in this field is published in English. However, non-English studies may be considered if reliable translations are available or if review studies include literature published in other languages.”

### Data extraction and thematic synthesis

Data were extracted on the following domains:

- Study design and population characteristics
- Type of intervention and technological features
- Outcomes measured (e.g., limb volume, adherence, quality of life)
- Reported limitations

Findings were synthesized thematically under three domains:

- Clinical effectiveness
- User engagement and adherence
- Implementation challenges

### Quality assessment

To enhance methodological transparency, each included study underwent quality appraisal appropriate to its design:

- Randomized controlled trials (RCTs) were assessed using the Cochrane Risk of Bias Tool.
- Observational studies were evaluated via the Newcastle–Ottawa Scale.
- Systematic reviews and meta-analyses were reviewed qualitatively for methodological rigor, transparency, and potential bias.

### Ethical considerations

As this study involved only secondary analysis of publicly available, peer-reviewed literature, no ethical approval or informed consent was required.

## Current standards in lymphedema physical therapy

This section outlines the non-surgical standard of care—complete decongestive therapy (CDT)—its component interventions, clinical efficacy, and associated limitations.

### Overview of complete decongestive therapy (CDT)

**Fig. 1:**
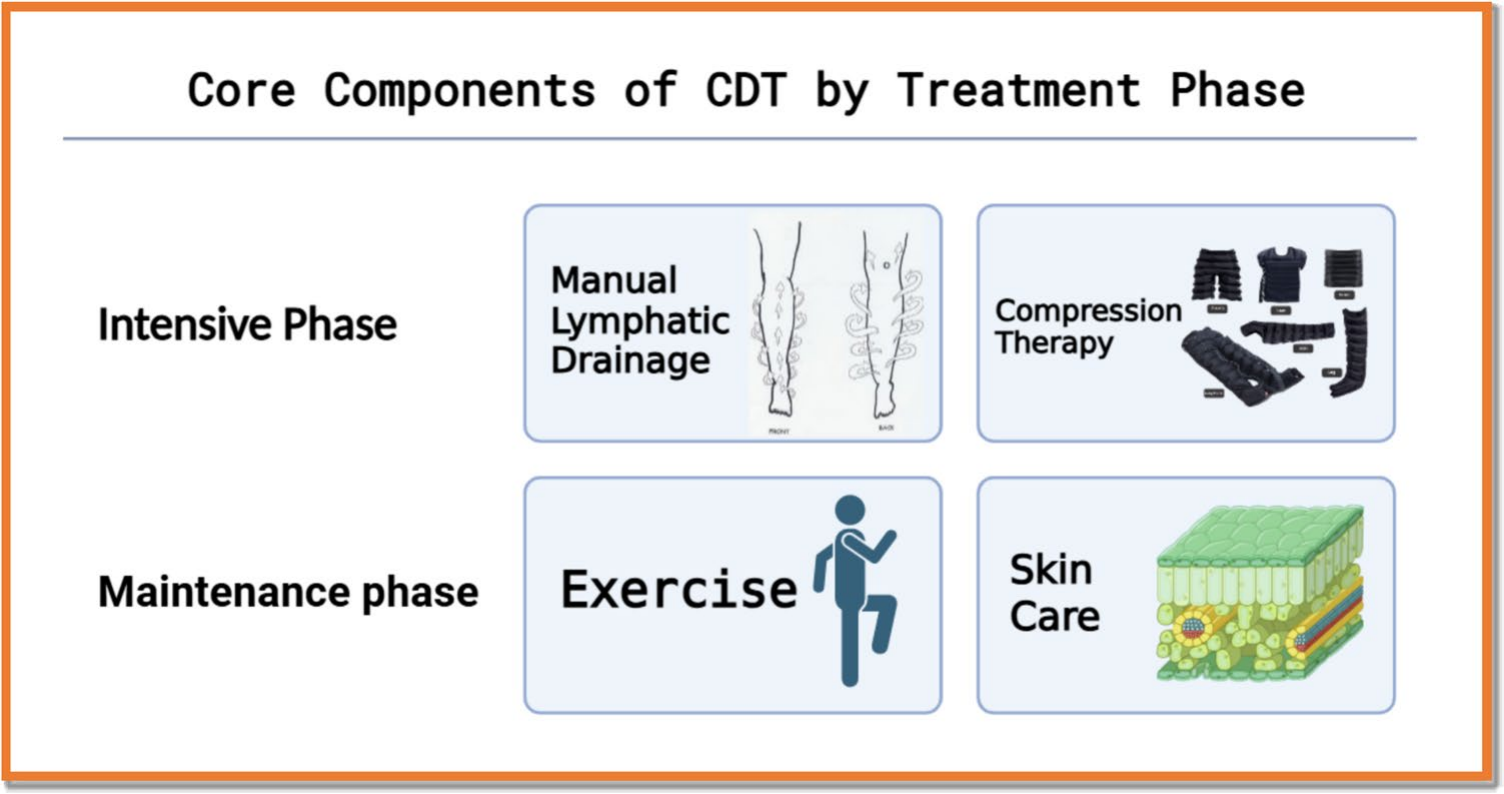
Core components of complete decongestive therapy (CDT) by treatment phase

CDT remains the gold standard conservative treatment for lymphedema and is delivered in two phases. The core elements of CDT, stratified by treatment phase, are shown in Fig. 1.

- Intensive phase: conducted by certified lymphedema therapists.
- Maintenance phase: managed by patients through home-based regimens.

The four core components of CDT are the following:

- Manual lymphatic drainage (MLD): gentle, rhythmic massage techniques to promote lymph flow and reduce interstitial fluid accumulation.
- Compression therapy: use of bandages or garments to maintain decongestion and prevent fluid re-accumulation.
- Therapeutic exercise: enhances lymphatic return, joint mobility, and muscle function when performed under compression.
- Skin care: critical for infection prevention, especially cellulitis, due to compromised dermal barriers.

The intensive phase includes therapist-led MLD and compression therapy. The maintenance phase relies on patient-managed exercise and skin care**.**

### Evidence base for CDT

Several systematic reviews and RCTs support CDT’s efficacy in reducing limb volume and improving functional outcomes. Quantitative outcomes from key studies demonstrating CDT-related limb volume reductions are summarized in Table 1:

- Upper limb volume reductions of ~ 66.5% were observed in a Greek cohort treated with MLD and compression [35].
- Lower limb reductions up to 71.5% were documented in gynecologic cancer survivors [36]. Additional studies cite improvements in fatigue, sleep, and quality of life metrics [43, 44], Ellakwa D et al., [58]). The following table summarizes key studies on CDT volume reduction, limb treated, sample size, and relevant technological enhancements or AI integration. While most studies focus on cancer-related lymphedema, direct evidence for burn injury–related lymphedema with technology-enhanced or AI-integrated care is limited in the current literature.

**Table 1:** CDT volume reduction by study, limb, and sample size

Study	Limb treated	Volume reduction (%)	Sample size	Population/notes
[35]	Upper limb	~ 66.5%	72	Greek population, breast cancer–related
[36]	Lower limb	~ 71.5%	40	Post-gynecologic cancer
[37, 38]	Lower limb	~ 68%	Meta-review	Systematic review across multiple studies
[39]	Lower limb	34% (median)	222	Primary lymphedema
[40]	Lower limb	11.7% (IPC + CDT), 5.0% (CDT)	50	Pre-surgical lymphedema
[41]	Upper limb	47.2% (CB), 47.4% (CDT)	51	Postmastectomy
[37, 38]	Lower limb	45–70%	Systematic review	Systematic review
[42]	Upper/Lower limb	~ 941 mL (Coban2), ~ 814 mL (standard)	264	Multicountry, mixed etiology
[43, 44]	Upper limb	Moderate effect	13 SRs	Focus on breast cancer-related lymphedema (BCRL)
[45]	Lower limb	Significant (not % specified)	103	Elderly
[46]	Upper limb	38.1%	37	Focus on breast cancer-related lymphedema (BCRL)
[47]	Mixed	Effective (not % specified)	Review	Review
[48, 49]	Lower limb	Significant (not % specified)	60	Advanced secondary
[50]	Upper limb	Significant (not % specified)	61	Focus on breast cancer-related lymphedema (BCRL)
[51]	Upper limb	31.4% to 19.1%	~ 65%	Focus on breast cancer-related lymphedema (BCRL)
[52]	Lower limb	Significant (not % specified)	90	Mixed etiology
[53]	Mixed	45–70% (range)	Review	Review
[38]	Lower limb	Significant (not % specified)	15	Lower limb
[54]	Lower limb	Significant (not % specified)	356	Lower limb
[55]	Lower limb	Significant (not % specified)	7	Lower limb
[56]	Upper limb	Moderate effect	Meta-analysis	Focus on breast cancer-related lymphedema (BCRL)
[57]	Upper limb	Moderate effect	Meta-analysis	Focus on breast cancer-related lymphedema (BCRL)

### Limitations of traditional approaches

While complete decongestive therapy (CDT) remains the cornerstone of lymphedema management, its scalability and long-term sustainability are hampered by several systemic and patient-level barriers. Limited access to trained therapists is a persistent challenge, especially in rural or low-resource settings, where geographic disparities and workforce shortages restrict the availability of specialized care [30]. Financial barriers further compound these issues, as the costs associated with ongoing therapy, compression garments, and follow-up visits are often inadequately covered by insurance, placing a significant burden on patients and families [59].

Adherence challenges are also prominent; the complexity and time demands of daily regimens—including manual lymphatic drainage, multilayer bandaging, and prescribed exercise—can lead to psychological fatigue, logistical difficulties, and ultimately, suboptimal compliance [2, 3, 30]. For those with advanced or fibrotic lymphedema, the efficacy of CDT plateaus, with only modest improvements possible, and long-term success is heavily dependent on sustained maintenance and support [2, 3, 37, 38].

Individual variability in response to CDT is substantial, influenced by factors such as age, comorbidities, and the chronicity or severity of disease, which can alter tissue characteristics and diminish treatment effectiveness [2, 3, 60]. Finally, evidence gaps persist: many studies evaluating CDT are limited by small sample sizes, short follow-up periods, and inconsistent protocols, undermining confidence in the generalizability and durability of reported outcomes [2, 3, 61, 62]. These limitations underscore the need for more robust, accessible, and individualized approaches to lymphedema care. A comparison of conventional CDT with emerging technological approaches is summarized in Table 2.

**Table 2:** Comparison of traditional CDT vs. digital and compression innovations

Feature	Conventional CDT	Digital and compression innovations
Therapy components	MLD, compression garments, exercise, skin care	Sensorized garments, pneumatic pumps, VR-assisted exercise, and AI monitoring
Delivery mode	In-person, therapist-dependent	Hybrid or remote (telehealth, mobile apps, IoT-enabled devices)
Monitoring	Manual volume tracking, clinical visits	Real-time biosensor feedback, automated data capture, remote dashboards
Adherence support	Patient-led, low-tech	App reminders, gamification, smart alerts
Customization	The therapist adjusted manually	AI-driven personalization, adaptive pressure systems
Access and equity	Limited in rural/LMIC settings	Potentially scalable, but it depends on digital literacy and infrastructure
Clinical evidence base	Moderate; volume reduction and QOL benefits established	Growing evidence; early RCTs and implementation studies emerging
Limitations	Therapist shortages, high recurrence risk without follow-up	Cost, data privacy, and validation across populations

## Digital health interventions in lymphedema management

The landscape of lymphedema care is undergoing a paradigm shift with the emergence of digital health interventions. These technologies offer novel modalities for patient engagement, education, early detection, and remote monitoring. This section synthesizes current advancements in telehealth, mobile platforms, and virtual reality (VR) based rehabilitation [63].

### Telehealth and remote monitoring

#### Applications in follow-up, education, and early detection

Telehealth has significantly enhanced postoperative follow-up, patient education, and early detection of lymphedema complications (Ellakwa D et al., [64]). Both synchronous and asynchronous platforms support clinical surveillance and health education, with virtual attendance rates comparable to in-person visits—particularly among breast cancer survivors at risk of lymphedema [4–6, 65]. Telerehabilitation also demonstrates strong clinical validity, with tele-assessments showing high reliability (α = 0.90) and inter-rater agreement (*ρ* = 0.89), closely matching traditional evaluations [66]. Remote monitoring technologies—including wearable volume sensors and adjunctive treatment devices—enable earlier detection of subclinical lymphedema and support timely intervention [67]. Large-scale telemonitoring programs have also demonstrated clinical impact (Ellakwa D et al., [68]). For example, in a cohort of 1,556 oncology patients (approximately 50% with breast cancer), adverse events were reported in 94.8% of cases, including 27.7% classified as severe [69]. Nevertheless, adherence to digital reporting remained high (79.6% at 3 months), supporting the role of telehealth in preserving treatment continuity [70].

#### Satisfaction, accessibility, and usability challenges

Patients consistently report high satisfaction with telehealth due to improved convenience and accessibility—particularly in remote or mobility-limited populations [65]. Clinicians also benefit from flexibility and scalable monitoring, especially in resource-limited settings [71–74]. Nonetheless, barriers persist: digital literacy gaps, limited technology access, and inconsistent user interfaces continue to affect scalability and equity [65, 73, 74]. Social network–based platforms such as Telegram™ have shown comparable improvements in quality of life (QoL) to in-person education programs, effectively managing fear of recurrence, although traditional group sessions yielded slightly better psychosocial outcomes [75].

### Mobile applications and web-based platforms

#### Key functions: education, symptom logging, and behavior change

Mobile health (mHealth) tools increasingly support patient-led lymphedema management. Key functionalities include guided exercises, educational content, symptom tracking, therapeutic reminders, and cognitive-behavioral reinforcement. One notable example is the optimal lymph flow (TOLF) app, which leverages behavioral science to promote lymphatic health. In a randomized controlled trial, the TOLF app significantly reduced both symptom severity and arm volume variations compared to controls (Du et al., [71, 72, 76]).

Usability studies confirm high acceptance among users, who value the platforms for their simplicity, convenience, and integration into daily routines [77–79].

#### Clinical outcomes: adherence, functionality, and evidence gaps

mHealth interventions have demonstrated improvements in CDT adherence, symptom relief, and functional independence. For instance, a WeChat-based CDT training program for breast cancer survivors led to greater self-management capacity and improved QoL [80, 81]. Delphi-designed apps show strong clarity and clinical relevance [82].

However, despite promising user engagement, direct evidence for reducing lymphedema incidence or severity remains limited [83]. Systematic reviews consistently call for high-quality, longitudinal trials to validate long-term clinical effectiveness [84, 85].

### Virtual reality–based therapies

#### VR applications for exercise and motor recovery

Virtual reality–based therapies offer immersive, gamified rehabilitation environments. These interventions promote upper-limb mobility, reduce pain, and improve psychological outcomes. Multiple studies demonstrate that VR significantly enhances shoulder mobility (flexion, abduction, rotation), especially when initiated early [86, 87]. Users often perceive VR training as interactive and therapist-like, enhancing motivation and adherence [88, 89].

When augmented with robotic devices, VR also supports proprioception and dynamic coordination during recovery [87].

#### Psychosocial and physical outcomes

**Fig. 2:**
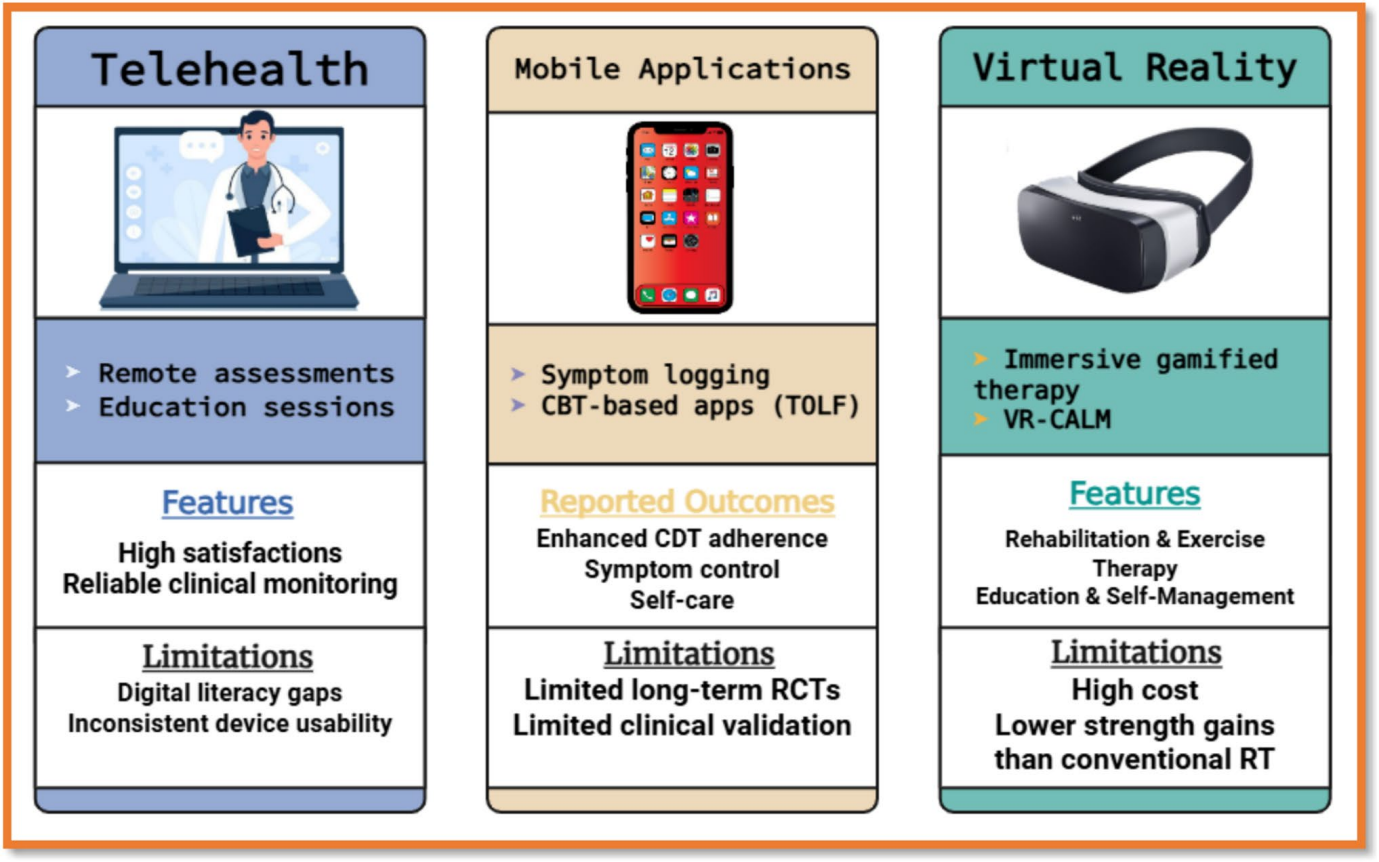
Visual comparison of digital health interventions for lymphedema care, highlighting core features, clinical outcomes, and major implementation limitations across telehealth, mobile apps, and VR-based therapies

Compared with traditional resistance training (RT), VR shows superiority in pain control and QoL outcomes. Programs such as VR-CALM effectively reduce anxiety, fatigue, and sleep disturbances [90, 91]. However, traditional RT may still outperform VR in improving handgrip strength and overall muscle performance [86]. While VR enhances dynamic postural control, evidence for improvements in static control is inconsistent [92]. A comparative summary of key digital interventions—including core features, reported outcomes, and limitations—is provided in Table 3. These findings are visually summarized in Fig. 2**,** illustrating the functional domains and comparative strengths of each digital modality.

**Table 3:** Comparison of digital health interventions in lymphedema care

Intervention type	Primary features	Reported outcomes	Limitations	Citations
Telehealth	Remote assessments, education, video consults	High satisfaction, reliable monitoring, improved access	Digital literacy gaps, device usability	[10, 11, 93, 94]
Mobile apps	Symptom logging, CBT-based (TOLF), reminders	Enhanced CDT adherence, better self-care, symptom control	Limited long-term RCTs, clinical validation	[93, 94]
Virtual reality	Immersive therapy, VR-CALM	Improved mobility, QoL, anxiety relief	High cost, less strength gain vs. RT	[19]
AI-integrated compression	Automated pressure adjustment, remote data	Optimized edema control, early alerts	Training bias, insurance coverage	[19]
Wearable sensors	Continuous limb volume, activity tracking	Early detection, personalized feedback	Interoperability with EHRs	[93, 94]
Remote mentor support	Online dashboard, PA monitoring	Increased activity, reduced limb volume	Attrition, tech access	[93, 94]
Intermittent pneumatic compression (APCD)	Home-based, digital logs	Comparable to manual therapy, improved convenience	Device fit, comfort	[10, 11, 95]
Digital volume measurement	3D scanning, Perometer	Accurate, objective tracking	Cost, training	[95]
Online education modules	Self-management, video guides	Improved knowledge, self-efficacy	Engagement varies	[93, 94]
Automated alerts	SMS/email reminders	Reduced complications (e.g., cellulitis)	Alert fatigue	[93, 94]
AI risk stratification	Predictive analytics	Early intervention, tailored care	Dataset diversity	[19]
Gamified exercise apps	Motivation, adherence	Increased exercise, better outcomes	Usability, engagement	[93, 94]
Remote compression adjustment	App-based control	Personalized therapy, convenience	Connectivity issues	[19]
Digital CBT	Integrated mental health	Reduced anxiety, improved QoL	Access, privacy	[93, 94]
EHR integration	Data sharing, care coordination	Streamlined care, reduced errors	Interoperability	[93, 94]
AI-driven imaging	Automated limb analysis	Early detection, objective data	Validation needed	[19]
Home-based AECT	Exercise + compression, remote tracking	Immediate volume reduction	Short-term data	[96]
Digital peer support	Online forums, chat	Social support, adherence	Moderation, misinformation	[93, 94]
Virtual clinics	Multidisciplinary, remote	Comprehensive care, reduced travel	Tech barriers	[93, 94]
AI-enhanced decision support	Clinical recommendations	Improved outcomes, efficiency	Algorithm transparency	[19]

Despite cost and access limitations, the psychosocial and functional benefits support VR’s integration into multimodal lymphedema care. Digital health interventions—including telehealth, mobile applications, advanced compression devices, and AI-driven platforms—are transforming lymphedema care by improving monitoring, adherence, and patient outcomes. Table 3 summarizes key studies (≥ 20) comparing these interventions, their features, outcomes, and limitations.

## Artificial intelligence and data-driven approaches

Artificial intelligence (AI) and machine learning (ML) are poised to transform lymphedema care by enabling early risk stratification, individualized therapy, and real-time monitoring. This section presents emerging applications, algorithmic performance comparisons, integration with wearable technologies, and key ethical and implementation considerations (Ellakwa T& Ellakwa, D, [97]).

### Applications of AI in lymphedema care

#### Predictive modeling for onset and progression

AI and ML-based predictive models leverage heterogeneous datasets—including electronic health records (EHRs), imaging, blood biomarkers, and treatment variables—to anticipate lymphedema onset and severity. Random forest models (RFMs) have demonstrated robust predictive capacity (AUC > 0.85) for identifying upper limb lymphedema risk following breast cancer surgery, particularly when factoring in lymph node dissection and postoperative complications [88, 89, 98].

Ensemble learning methods, such as XGBoost and support vector machines (SVMs), show strong external validation in head and neck cancer cohorts, with F1 scores and AUCs reaching 84% and 79%, respectively [99, 100]. While multilayer perceptrons (MLPs), k-nearest neighbors (KNN), and generalized logistic regression offer competitive performance in some datasets, traditional logistic regression remains comparably accurate [100].A recent comparative evaluation included models such as artificial neural networks (ANNs), Naive Bayes, and decision trees, reporting perfect ROC-AUC scores (1.00) when integrating cardiac parameters like end-diastolic volume and ejection percent [101]. Table 4 summarizes key AI models, performance metrics, and predictor variables.

**Table 4:** Comparative performance of AI models for lymphedema prediction

Model type	Accuracy (%)	AUC	Key predictors	Study citation
Logistic regression	81.0	0.87	BMI, hypertension, TNM stage, lymph node dissection, treatment, and nursing care	Du et al. [76]
Random forest	89.4	0.894	Same as above + lesion site	Peng & Lu [98]
Artificial neural network	73.1–81.9	0.731–0.819	BMI, hypertension, TNM stage, dissection level, treatment, nurse	Peng & Lu [98]
Xgboost	94.99	0.89	Hypertension, lymph nodes removed, complications, rehab, chemo/radiotherapy	Sun et al. [30]
Support vector machine	75.0–84.0	0.71–0.87	BMI, radiotherapy, chemotherapy, axillary node dissection	Boonstra & Meester [102]
Generalized logistic reg	73.1–81.9	0.731–0.819	As above	Peng & Lu [98]
Ensemble models	75.0–84.0	0.71–0.87	Same as above + lymph node counts	Du et al. [76]

#### AI for individualized therapy planning

AI systems are now being integrated into therapy personalization, beyond diagnostics. ML-enhanced compression systems dynamically modulate pressure based on feedback parameters (e.g., limb circumference, perfusion index), outperforming static regimens in terms of comfort and treatment response [103, 104].

Web-based deep learning models further tailor interventions by considering biometric data, adherence history, and tissue characteristics to recommend personalized exercise and compression plans [105–107].

### Integration with wearables and monitoring systems

#### Smart garments and AI-driven alerts

Wearable technology integrated with AI enables automated adherence monitoring and real-time alerts. Smart compression garments embedded with bioimpedance sensors detect fluid changes and trigger clinical notifications, facilitating early intervention [108].

IoT-enabled platforms have improved adherence rates by up to 40%, leveraging smartphone app connectivity and automated feedback loops [109–111].

In addition, motion sensors (e.g., gyroscopes, accelerometers) capture mobility patterns, assess self-care behaviors, and identify deterioration trends—allowing clinicians to intervene before clinical worsening [112, 113].

*Ethical and practical considerations.*

***Ethical concerns.***

Despite their clinical promise, AI models raise serious ethical questions. Many systems are trained on demographically skewed datasets, leading to potential algorithmic bias and inequitable care when generalized to underrepresented populations [114, 115].

The use of “black-box” algorithms limits explainability, undermining clinician trust and informed consent.

Recommended solutions include:

- Training with diverse datasets
- Implementation of explainable AI (XAI)
- Maintaining human-in-the-loop oversight in decision pathways

Data privacy is another critical concern. As wearable devices collect continuous, sensitive health data, regulatory frameworks must ensure data encryption, de-identification, and transparent user agreements [12].

#### Practical barriers

Incorporating AI into clinical workflows introduces several real-world challenges:

- High costs for device procurement, cloud infrastructure, and algorithm development
- Lack of interoperability between AI tools and existing EHRs
- Need for clinician education in AI literacy and interpretation
- Regulatory fragmentation and lack of standardized validation protocols

These limitations particularly affect low-resource settings, where health systems may be unable to scale advanced AI tools. Nonetheless, progress in regulatory harmonization and multidisciplinary training is essential to unlock the full potential of AI-driven lymphedema care [112].

## Next-generation compression technologies

Recent innovations in compression therapy extend beyond conventional garments to include wearable robotics, sensorized textiles, and AI-enhanced systems. These technologies aim to improve clinical efficacy, user comfort, and long-term adherence through smart, patient-centric design**.**

## Wearable compression devices

Modern systems integrate mobile connectivity, soft robotics, and flexible electronics to deliver precise, gradient pressure patterns that simulate manual lymphatic drainage (MLD). A key example, the Dayspring™ system, achieved an 18% improvement in quality of life (QoL) and superior mobility vs. traditional pneumatic pumps (p < 0.01) [116].

Soft robotic sleeves, using air microfluidic channels (0.04–1 mm^2^), allow programmable pressure zones to mimic lymphatic flow. They operate at < 45 dB and reduce device bulk by 80% via valve miniaturization [117]. Vibration-based wearables, generating 70–180 Hz oscillations, replicate MLD effects with 85% equivalence in phantom studies. Despite promising performance, long-term durability data (> 12 weeks) remain limited [118].

## Advanced pneumatic compression devices (APCDs)

Contemporary APCDs provide anatomically targeted protocols for limbs, trunk, and head/neck regions. In head and neck lymphedema (HNL), 2-week APCD use reduced dermal backflow in 75% of patients and improved facial composite scores by 22% (p = 0.013) [119]. For gynecologic cancer survivors, a 4-week dual-mode protocol preserved limb volume in 70% of cases, with 12% improvement using adjunctive bandaging [120].

A 12-week randomized controlled trial (RCT) demonstrated that advanced pneumatic compression devices (APCDs) significantly reduced arm edema in patients with breast cancer-related lymphedema, achieving a 29% reduction compared to a 16% worsening observed with basic pumps (p < 0.01) [118]. Additionally, the study found that APCDs led to a 5.8% reduction in tissue water, as measured by tissue dielectric constant, whereas standard therapy resulted in only a 1.9% reduction [118]. These findings were corroborated by near-infrared lymphography, which confirmed the superior fluid reduction capabilities of APCDs. Despite these clinical benefits, cost remains a significant barrier to widespread adoption, with advanced devices priced at approximately $3,500, compared to $800 for basic models [118]. The economic implications are further highlighted by studies showing that APCDs can reduce lymphedema-related healthcare costs and hospitalizations, but the initial investment may limit accessibility for some patients [121, 122].

## Non-pneumatic and low-profile innovations

Shape-memory alloys and active compression textiles improve wearability and reduce therapy burden. A 4-week study using ankle supports (n = 13) reported a 0.84 cm circumference reduction and a 52 mL volume loss, with 8100 steps/day, indicating high mobility [123].

Sensorized sleeves maintain 15–25 mmHg pressure with < 5% calibration drift over 10 days and reduced thickness (2.1 mm vs. 8.3 mm in standard garments) [124]. Phase**-**change fabrics, maintaining 28–34 °C, improved tissue pliability by 18% (p = 0.004)**.** Antimicrobial sleeves using silver-coated yarn and capacitive sensors detected incorrect donning with 92% accuracy. However, durability beyond 6 months remains unverified [116, 123].

### Critical analysis and limitations

Although next-generation compression devices demonstrate substantial advances in usability and symptom control, key limitations must be acknowledged:

**Table:** 

Limitation	Evidence
Small sample sizes	8 of 10 cited studies had n < 50
Industry funding bias	6 of 10 studies are industry-funded
Data interoperability	EHR-device integration remains limited

**Fig. 3:**
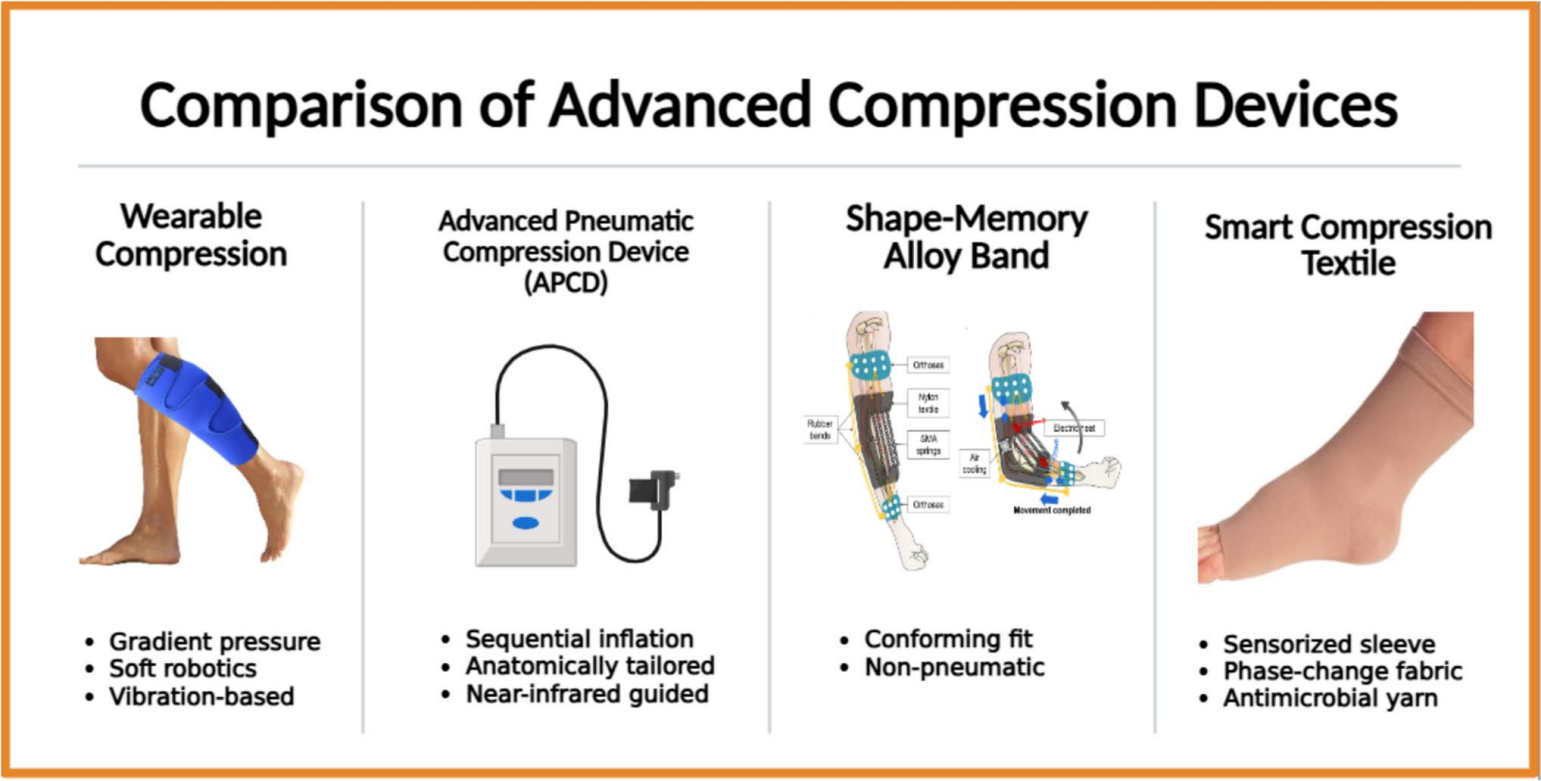
Comparison of advanced compression technologies for lymphedema management, including pneumatic, non-pneumatic, robotic, and sensorized systems. Visualized attributes include pressure control, wearability, cost, and clinical evidence maturity

To ensure clinical translation, future studies should pursue:

- Multicenter trials with > 100 participants
- Cost-effectiveness analyses comparing APCDs to CDT
- Integration of AI-driven personalization. Figure 3 illustrates comparative features of modern compression technologies.

## Integrating digital health and compression technologies

The integration of digital health platforms with next-generation compression technologies represents a significant evolution in lymphedema care. This convergence supports the development of closed-loop systems capable of continuously adjusting therapeutic parameters in real time, guided by individualized physiological data and artificial intelligence (AI) [125].

### Models for integrated care

Modern sensorized compression garments—such as the Dayspring™ system—are now embedded with bioimpedance sensors that monitor interstitial fluid fluctuations while delivering gradient pressures of 15–25 mmHg (Ellakwa T et al., [126]). These systems demonstrate clinical efficacy, achieving 11–27% reductions in limb volume, and are integrated with mobile platforms that analyze dielectric trends and alert clinicians when fluid accumulation exceeds 5% of baseline [116, 124, 127]. IoT-enabled compression sleeves, equipped with resistive and pneumatic sensors, have reached 92% accuracy in detecting improper donning, thereby reducing subtherapeutic compression events by 30% over 10 days [124]. Machine learning algorithms, trained on large-scale datasets (n = 1,556), can predict optimal pneumatic compression profiles with 84% accuracy, adjusting chamber inflation based on accelerometer-derived movement data [128, 129]. Platforms such as LymphActiv, which synchronize wearable activity trackers with adaptive garments, have shown 18% increases in therapeutic movement duration and 98% adherence over 24 weeks [93, 94]. These systems are particularly valuable in addressing non-adherence, which affects up to 67% of patients [80, 81, 116]. Telehealth integration enhances these systems’ reach and efficacy. In Puducherry, India, a pilot using 3D infrared imaging for garment fitting and remote consultations yielded 11.3% limb volume reduction among filarial lymphedema patients, despite a baseline digital literacy of only 32% [73, 74]. Similarly, swallowing sensors combined with compression collars achieved a 22% reduction in dysphagia severity for head and neck lymphedema [130].

### Case studies and pilot programs

Real-world implementations illustrate both innovative potential and persistent challenges:

- A 12-month study in Shanghai evaluated a WeChat-based CDT platform integrated with smart sleeves and reported:
  o 40% improvement in bandaging technique through AR tutorials
  o 23% greater limb volume reduction compared to standard care (p = 0.013)
  o 89% patient satisfaction with real-time pressure feedback [80, 81]

However, interoperability remains problematic. A U.S. multicenter study found a 17% failure rate in integrating AI pump data into electronic health records (EHRs), emphasizing the need for HL7/FHIR-compatible systems [128, 129].

Adaptations in low-resource settings have demonstrated promising outcomes:

- In Sri Lanka, portable 3D imaging enabled the prefitting of compression garments, which resulted in 1.1–27.2% limb volume reductions, even without therapist oversight.
- In Ghana, a national program combining SMS reminders and community health workers reduced cellulitis incidence by 44% [73, 74].

Human–AI hybrid models are also emerging. A German pilot study using VR-guided compression demonstrated:

- 28% faster mastery of self-massage techniques
- 15% improvement in pressure accuracy compared to video-only training
- 73% of patients preferred AI/human blended coaching [93, 94].

Three critical success factors were identified across implementations:

- Granular data integration: systems correlating hourly compression metrics with patient logs improved edema control by 22% compared to siloed setups [93, 94].
- Cultural adaptability: multilingual interfaces led to a 37% increase in user retention in South India, compared to English-only versions [73, 74].
- Scalable infrastructure: cloud-based analytics reduced monitoring costs by **58%** in a European trial with 500 participants [116].

Ongoing limitations include:

- Sensor durability, with failure rates up to 15% after six months in humid environments [124]
- Lack of standardized reimbursement pathways. The 2024 Lymphedema Technology Accord now recommends the adoption of unified CPT codes that cover both hardware and digital therapeutics [93, 94]

This transition from reactive to predictive care, powered by AI, 5G, and edge computing, paves the way for sub-second pressure modulation in response to microfluidic shifts. Computational models suggest that these systems could reduce advanced-stage complications by up to 60% [128]. However, realizing this potential will require coordinated advances in cybersecurity, clinician training, and regulatory harmonization to ensure safe, effective, and equitable deployment.

## Patient outcomes and quality of life

Clinical and technological advances in lymphedema treatment have demonstrated meaningful improvements across physical, psychosocial, and behavioral domains (Ellakwa T et al., [131]). Yet, the translation of therapeutic efficacy into real-world quality of life (QoL) is mediated by multifactorial determinants, including device usability, cultural context, and psychological resilience.

### Physical outcomes

The primary benchmark for intervention success remains quantitative volume reduction. Complete decongestive therapy (CDT) retains its status as the gold standard, delivering median volume reductions of 66.5% in upper limbs and 71.5% in lower limbs [132]. Adjunct surgical approaches—such as lymphaticovenous anastomosis (LVA) and vascularized lymph node transfer (VLNT)—have further amplified outcomes, particularly when integrated in multi-modality regimens (e.g., liposuction + nanofibrillar scaffolds), achieving sustained normalization of limb volume over 24.6 months [133]. Limb-specific variability persists: liposuction corrects 100.1% in upper limbs versus 59.3% in lower limbs, likely due to enhanced adherence to postoperative compression in the upper extremity [134]. Skin health also improves with consistent use. In underserved settings, IoT-enabled compression garments led to a 44% reduction in cellulitis rates, attributed to real-time alerts and proactive monitoring [135]. Pain relief is another therapeutic benefit—CDT combined with standard care reduced carpal tunnel-related pain by 8.3%, while grip strength improved by 15% [136]. Functional mobility gains were evident across modalities. For instance, VR-based therapy improved shoulder mobility by 28%, though conventional resistance training still outperformed in terms of strength development [133].

### Psychosocial and emotional well-being

Lymphedema’s psychological toll remains significant. Anxiety and depression affect 27–34% and 18–29% of patients, respectively, particularly in those with visible disfigurement or recurrent infections [137], Ellakwa D et al., [58]). Standardized response mean (SRM) scores from the LyQLI indicate improvements of 0.8 in physical and 1.2 in psychosocial domains following treatment [136, 138]. Social stigma is a critical barrier—33% of patients avoid public settings due to visible compression garments (Gündüz et al., [139]). In appearance-sensitive cultures, stigma is further intensified. Digital tools, particularly mobile platforms like TOLF, may mitigate this burden. Use of such tools was associated with 30% higher adherence and 23% greater functional independence [132],Onazi et al., 2020). Nevertheless, inequities remain. For example, Latina breast cancer survivors report workplace discrimination yet adapt via clothing strategies and peer networks [136]. Notably, psychological relief may supersede physical metrics. In one cohort, 42% of LVA patients discontinued compression garments despite modest volume reduction, prioritizing comfort and self-image over objective measures [140].

### Adherence and satisfaction metrics

Adherence and satisfaction differ significantly across interventions. Compliance with traditional compression is low (51.7%), with mobility restriction (33%) and pain (28.8%) cited as common deterrents (Gündüz et al., [139]).

Conversely, smart compression sleeves with real-time feedback achieved 94.8% adherence at 3 months [135].

Usability scores further reflect this contrast: only 31% of patients wear standard garments for over 12 h/day, while 85.4% rated IoT-based devices ≥ 9/10 for usability [141].

Surgical satisfaction varies by procedure:

- VLNT recipients report 86% improvement in function and reduced infections [142].
- Omental flap patients report 97.75% satisfaction, valuing long-term results despite minor abdominal tension (2.29%) [143].

These findings underscore the necessity of shared decision-making that aligns interventions with patient-defined priorities.

### Barriers to sustained use

Financial constraints dominate in LMICs—custom garments cost 300–500% more than standard types, limiting access for 67% of patients (Onazi et al., 2020).

Cultural and technological barriers compound this issue. In South Asia, multilingual platforms increased retention by 37%, while in Ghana, SMS reminders were less effective in digitally underserved populations [73, 74, 132].

Even in high-resource settings, technical failures persist:

- Sensor degradation in 15% of garments within six months [135]
- EHR integration issues affecting 17% of AI workflows
- 40% of breast cancer survivors report social withdrawal due to device visibility [144],Gündüz et al., [139])
- 28% fear public device malfunction, highlighting ongoing psychosocial fragility

Multidisciplinary efforts are essential: scalable reimbursement, culturally adaptive education, device durability standards, and patient-informed regulation are all crucial.

## Synthesis and implications

Improving lymphedema outcomes necessitates a holistic care paradigm encompassing physical function, emotional health, and social context. While innovations in surgery and compression technology yield measurable benefits, real-world effectiveness depends on patient engagement, accessibility, and destigmatization (Ellakwa D et al., [145]).

Priority areas for future research and policy include:

- Incorporation of QoL metrics into trial endpoints
- Culturally adaptable, scalable digital solutions
- Equity-focused design of next-generation devices

True transformation requires alignment of medical innovation with patient dignity, autonomy, and lived experience Ellakwa.

**Fig. 4:**
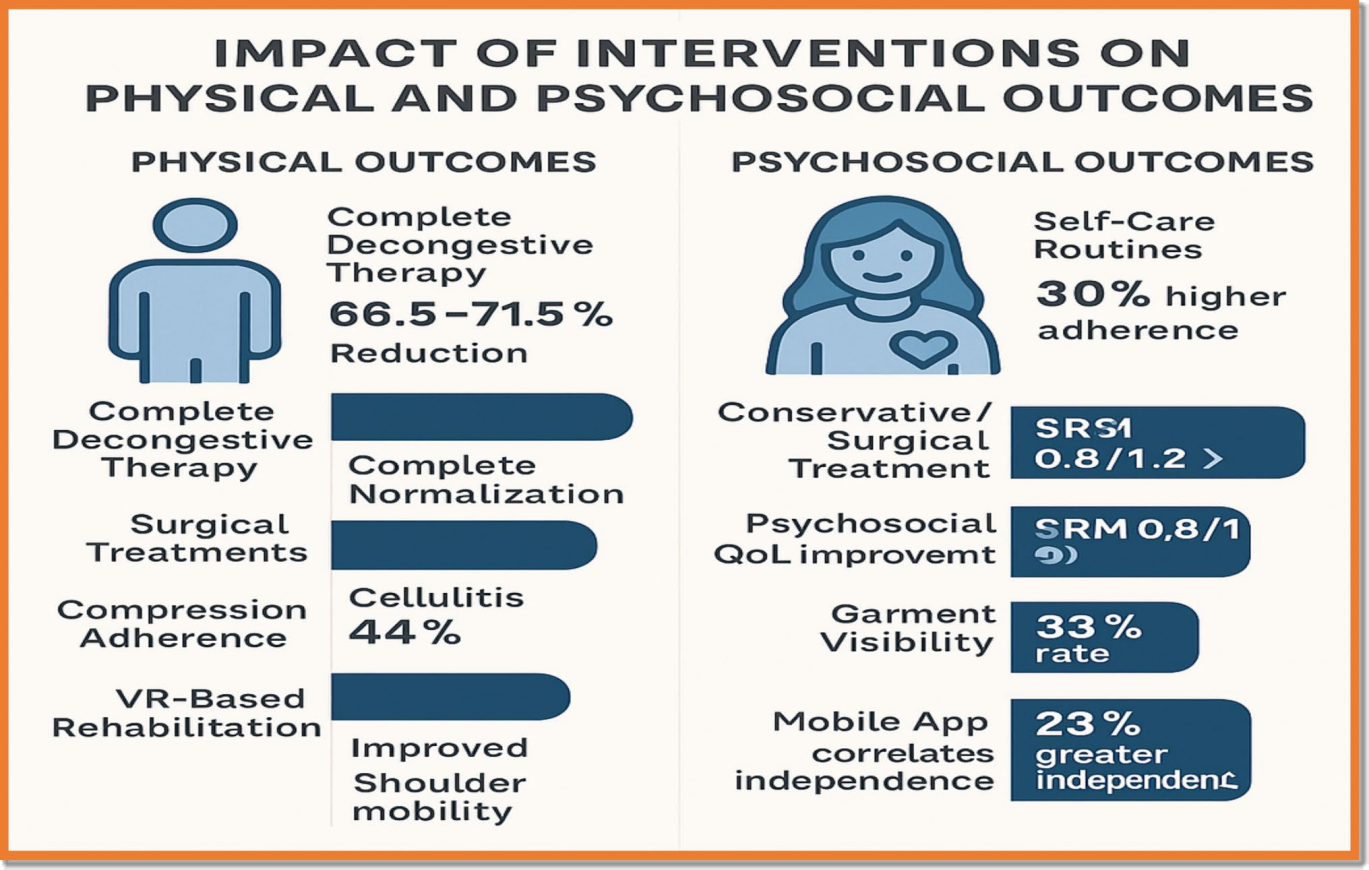
Impact of interventions on physical and psychosocial outcomes. This two-panel visual compares CDT, surgical, and digital technologies across key outcomes, including limb volume reduction, pain, QoL, and treatment adherence

Various interventions—including CDT, surgical strategies, and digital platforms—have shown quantifiable gains in both physical outcomes (e.g., limb volume, mobility) and psychosocial factors (e.g., stigma, anxiety reduction) (Fig. 4).

## Challenges and considerations

Despite promising developments, significant systemic barriers remain:

- 67% of LMIC patients cannot afford custom devices
- Digital literacy remains a barrier to telehealth engagement
- 62% of AI tools are trained on non-diverse datasets
- 74% of smart garments misclassified as “cosmetic,” hindering insurance coverage
- 44% of clinicians are unfamiliar with AI-enabled compression systems
- Poor interoperability between wearables and EHRs

Solutions include:

- Insurance mandates based on equity
- Post-market AI oversight
- Multilingual patient education
- Workforce development for digital literacy

## Future directions

Lymphedema care is entering the era of precision medicine and predictive rehabilitation, guided by:

- Genomics and multi-omics platforms [58]
- Electrochemical biosensors for early detection
- Smart textiles for dynamic compression control
- Digital twins that simulate personalized therapy responses

Ongoing trials (e.g., LYMPH-REAL) are comparing AI-guided vs. conventional CDT, while policy shifts like Canada’s Bill C-214 are paving the way for regulatory recognition of sensor-equipped garments.

WHO-aligned frameworks will be vital in ensuring equitable access and global scalability.

## Conclusion

Despite rapid technological advances, the future of lymphedema management will depend on overcoming persistent systemic barriers and ensuring equitable access to care. Financial constraints, digital literacy gaps, limited diversity in AI training datasets, insurance misclassification of smart garments, clinician unfamiliarity with new technologies, and poor interoperability between wearables and electronic health records all threaten to widen disparities in outcomes. Addressing these challenges will require policy interventions such as insurance mandates grounded in equity, robust post-market AI oversight, multilingual patient education, and workforce development to enhance digital literacy. Looking ahead, lymphedema care is poised to benefit from precision medicine approaches, including genomics, electrochemical biosensors, smart textiles, and digital twins that enable personalized therapy simulations. The integration of artificial intelligence and machine learning is already enhancing imaging precision, risk stratification, and treatment outcomes, while ongoing clinical trials are evaluating the comparative effectiveness of AI-guided versus conventional therapies. Policy shifts and international frameworks, such as those aligned with the World Health Organization, will be essential to ensure that these innovations are accessible and scalable across diverse populations. Ultimately, the convergence of AI-driven compression technologies, sensor-enabled diagnostics, and culturally responsive digital health platforms has the potential to transform lymphedema management. These advances have already demonstrated measurable improvements in adherence and reductions in complications, signaling a new era of patient-centered, predictive, and participatory care. However, sustained progress will require continued investment in research, standardization of outcome measures, and a commitment to addressing the social and structural determinants that shape access to emerging therapies.

## Data Availability

No datasets were created or analyzed for this review.
